# Prevalence and Sociodemographic and Lifestyle Determinants of Anemia during Pregnancy: A Cross-Sectional Study of Pregnant Women in China

**DOI:** 10.3390/ijerph13090908

**Published:** 2016-09-13

**Authors:** Xianglong Xu, Sheng Liu, Yunshuang Rao, Zumin Shi, LianLian Wang, Manoj Sharma, Yong Zhao

**Affiliations:** 1 School of Public Health and Management, Chongqing Medical University, Chongqing 400016, China; xianglong1989@126.com (X.X.); ls19940904@163.com (S.L.); 2 Research Center for Medicine and Social Development, Chongqing Medical University, Chongqing 400016, China; 3 Collaborative Innovation Center of Social Risks Governance in Health, Chongqing Medical University, Chongqing 400016, China; 4 School of Nursing, Chongqing Medical University, Chongqing 400016, China; rys0606@163.com; 5 Faculty of Health Sciences, School of Medicine, The University of Adelaide, North Terrace, Adelaide, SA 5005, Australia; zumin.shi@adelaide.edu.au; 6 The Department of Obstetrics, The First Affiliated Hospital of Chongqing Medical University, Chongqing 400016, China; llian_w@163.com; 7 Department of Reproduction Health and Infertility, The First Affiliated Hospital of Chongqing Medical University, Chongqing 400016, China; 8 Canada-China-New Zealand Joint Laboratory of Maternal and Fetal Medicine, Chongqing Medical University, Chongqing 400016, China; 9 Department of Behavioral and Environmental Health, Jackson State University, Jackson, MS 39213, USA; manoj.sharma@jsums.edu

**Keywords:** sociodemographic, lifestyle, anemia, pregnant women, China

## Abstract

Objective: This study aimed to assess the differences regarding anemia among pregnant women with diverse characteristics and lifestyle factors. Methods: A cross-sectional study of pregnant women was conducted between June and August 2015 in 16 hospitals in five provinces of Mainland China. Self-reported doctor-diagnosed anemia was used in the study. Results: We included 2345 pregnant women. Of the participants, 1755 (74.8%) were pregnant women of first pregnancy (PWFP) and 590 (25.2%) were second pregnancy (PWSP). The mean age of the participants was 28.1 years (SD 4.1). Overall, the prevalence of anemia was 12.7% (13.4% and 10.7% among PWFP and PWSP, respectively). The prevalence for not eating breakfast was 11.0%. Compared with PWFP, PWSP was inversely associated with the risk of anemia (odds ratio (OR) 0.66, 95% CI 0.48–0.91). Compared with those being registered in a low ranking hospital, pregnant women who were admitted to a high (OR 0.40, 95% CI 0.28–0.57) or a medium ranking hospital (OR 0.58, 95% CI 0.37–0.92) were inversely associated with the risk of anemia. Compared with women of low income (<¥4,500), those with high income were less likely to have anemia (OR 0.68, 95% CI 0.50–0.94). Compared with women with non-manual jobs, women with manual jobs (OR 1.70, 95% CI 1.17–2.45) and unemployed women (OR 1.42, 95% CI 1.04–1.93) were associated with a greater likelihood of suffering from anemia. Conclusions: Pregnant women not eating breakfast are of concern. Anemia is highly prevalent among pregnant women in China. Lower socio-economic status, manual jobs, PWFP, and those who attend a lower quality hospital have a greater likelihood of suffering from anemia. Tailored interventions are needed to address these issues.

## 1. Introduction

Anemia is linked to fatigue, weakness, dizziness and drowsiness [1]. Maternal anemia is associated with increased risk of miscarriages, stillbirths, prematurity and low birth weight of the infant, neonatal and maternal mortality [2]. Failure to reduce anemia worldwide has impaired the health and quality of life of millions of women, leading to generations of children with impaired development and learning, and communities and nations with impaired economic productivity and development [3]. More than 40% of pregnant women are anemic worldwide [4]. Globally, the prevalence of anemia in South Asia and Central/West Africa was the highest [5]. The prevalence of anemia fell from 33% to 29% in non-pregnant women, and from 43% to 38% in pregnant women between 1995 and 2011 [6]. In 2011, 29% of non-pregnant women were anemic [7]. According to the 2015 Chinese Chronic Diseases and Nutrition Report, the prevalence of anemia among Chinese adults aged 19 to 44 was 10.2% in 2012 [8].

Studies in many countries have reported that various factors are associated with anemia during pregnancy. A study in the Congo showed that maternal anemia was associated with pre-pregnancy high-risk factors such as age (<18 or  ≥35 years), previous miscarriage, high multiparity, diabetes in the family, previous prematurity and previous cesarean section; all enhanced the link of maternal anemia and complications [9]. In Uganda, compared with the first trimester (14.6%) and second trimester (20.7%), the prevalence of anemia during the third trimester was highest (24.3%) [10]. A study in Beijing showed that the incidence of anemia among pregnant women of first pregnancy (PWFP) and pregnant women of second pregnancy (PWSP) was 2.68% and 10.34%, respectively [11]. Cultural beliefs on food taboo for pregnant women contribute to the incidence of anemia in many countries [12]. Physical inactivity can increase the risk of many diseases/disorders [13]. Greater screen time duration was associated with lower overall health related quality of life [14]. Individuals who have insufficient sleep were more likely to suffer from chronic diseases such as cardiovascular disease, diabetes, depression, or obesity [15]. These health problems often result in poor dietary habits which may lead to anemia.

The determinants of anemia during pregnancy have not been studied thoroughly. Clarifying the factors that affect anemia during pregnancy is necessary to reduce this condition in China. Considering the different cultural backgrounds and social systems, findings from other countries may not be applicable in China. Previous study has shown that a comprehensive approach is needed to help anemic pregnant women improve maternal and child well-being [7].

On 29 October 2015, China reversed its One Child policy, a birth control policy that had lasted for 38 years since 1978, and now allows married couples to have a second child. In view of this, this study aimed to assess the differences of anemia among pregnant women with diverse characteristics and lifestyle factors including not eating breakfast, the average daily exercise and sleep time, and screen time (time spent viewing TV and using computers).

## 2. Materials and Methods

### 2.1. Research Method

The study design and methods, including the population and sample and the sampling framework, as well as the survey administration, pilot study and questionnaire development process have been reported previously [16,17]. All pregnant women visiting 16 hospitals in Chongqing, Chengdu, Zunyi, Liaocheng, and Tianjin between June and August 2015 were invited to participate. Chongqing, Chengdu, and Zunyi are in South China, whereas Liaocheng and Tianjin are in North China. The participants were interviewed in a hospital while they waited to receive prenatal care. A total of 2400 women participated in the study with a response rate of 97.76% (2400/2455). Among 2400 respondents, the final analysis sample included the 2345 persons who answered all questions. All participants provided written consent. The study protocol was approved by the Ethics Committee of Chongqing Medical University (record number 2015008). 

#### 2.1.1. Sociodemographic Variables

Demographic data included age (recoded as 18–25, 26–35 and 36–45 years), residence (urban/rural), per capita household income (recoded as low <¥4,500, medium ¥4,500–9,000 and high: >¥9,000), occupation (Rural migrant workers/Urban and rural unemployed, unemployed/Industrial workers of Non-agricultural registered permanent residence/Individual business/Business services staff/Civil servants/Senior manager and Middle-level manager in large and medium enterprise/Private entrepreneur/Professionals/Clerks/Students/Others), education level (low ≤ primary school or junior middle school, medium-high school and high-university), only child status (yes/no), status of husband as an only child (yes/no), and marital status (Married and Unmarried/Divorced/Widowed). Pregnancy was divided into three stages (Early pregnancy/Mid-pregnancy/Late-stage pregnancy). Based on the official Chinese classification system for hospitals [18], hospital capacity/quality rank was categorized as high (3A), medium (2A) and low (2B and below).

In the multivariable analysis for anemia determinants, employment status was categorized as manual (rural migrant workers/industrial workers of Non-agricultural registered permanent residence/Business services staff), non-manual (individual business/civil servants/senior manager and middle-level manager in large and medium enterprise/private entrepreneur/professionals/clerk/students), unemployed, and others.

#### 2.1.2. Lifestyle Factors

Lifestyle factors used in the study included: average daily exercise time (insufficient exercise/sufficient exercise); average daily exercise duration of less than 30 min was defined as insufficient exercise. Sleep duration (insufficient sleep time/sufficient sleep time), insufficient sleeping duration was defined as sleeping less than seven hours. Time spent on TV (more than 2 h/less than 2 h), time spent on computer (low <2 h, medium 2–4 h and high >4 h), eating breakfast daily (yes/no), and smoking status (smoker/non-smoker).

#### 2.1.3. Outcome Variable

Anemia status and other health conditions were assessed by the question “Have you ever been told by a doctor or other health professional that you have (disease or condition)? (yes/no)”.

### 2.2. Data Analyses

The characteristics of the participants were summarized using frequencies and percentages, and presented using descriptive analysis. Chi-square tests were employed for comparisons when appropriate. Logistic regression analysis was used to assess the factors associated with anemia among pregnant women. The full logistic regression model adjusted for parity, single-child status, status of husband as a single-child, age, marital status, education level, residence, income, stages of pregnancy, job status, not eating breakfast, average daily exercise time, sleep duration, and time spent on TV and/or computers. All statistics were performed using 2-sided tests, and statistical significance was considered at *p* < 0.05. All data analyses were performed using a statistical software (SAS version 9.1; SAS Institute, Cary, NC, USA).

## 3. Results 

### 3.1. Characteristics of Participants

We included 2345 pregnant women (1755 PWFP and 590 PWSP). The mean age of the participants was 28.1 years (SD 4.1). The prevalence of not eating breakfast was 11.0%. Table 1 shows the characteristics of participants, as well as the prevalence of anemia by sociodemographic factors. Overall, the prevalence of anemia was 12.7% (13.4% in PWFP and 10.7% in FWSP). Among those attending a low rank hospital, the prevalence of anemia was 24.8%.

### 3.2. Factors Associated with Anemia in Logistic Regression Model

Compared with PWFP, PWSP was inversely associated with anemia (OR 0.66, 95% CI 0.48–0.91) (Table 2). Compared with those being registered in a low ranking (2B or below) hospital, pregnant women who were admitted to a high (OR 0.40, 95% CI 0.28–0.57)) or medium ranking hospital (OR 0.58, 95% CI 0.37–0.92) were less likely to have anemia. Women from medium income families were less likely (OR 0.68, 95% CI 0.50–0.94) to have anemia than those from low-income families. Compared with women with non-manual jobs, women with manual jobs (OR 1.70, 95% CI 1.17–2.45) and unemployed women (OR 1.42, 95% CI 1.04–1.93) were more likely to have anemia. Although, average daily exercise time, sleep duration, time spent on TV and computers included in the logistic regression model, and however they were not significantly associated with anemia.

## 4. Discussion

This study found that the prevalence of anemia was 12.71% (13.39% and 10.68% among PWFP and second pregnancy respectively). According to the *2015 Chinese Chronic Diseases and Nutrition Report*, the prevalence of anemia among adults aged 19 to 44 in 2012 in China was 10.2% [8]. In general, the prevalence of anemia among pregnant women is higher than the general population. Achieving a 50% reduction in the prevalence of anemia among women of reproductive age by 2025 requires a yearly reduction of 6.1% in this group [6]. Further measures are needed to reach the World Health Assembly target of a 50% reduction of anemia among women of reproductive age by 2025 [3]. Tailored interventions are also needed to reduce anemia during pregnancy in China.

An interesting finding of the study is the low prevalence of anemia among those in their second pregnancy. The finding is different from a previous study in China, which showed that the incidence of anemia among PWFP and PWSP was 2.68% and 10.34%, respectively [11]. We hypothesize that PWSP may be more familiar with knowledge about anemia prevention and pay more attention to the prevention of anemia. Compared with pregnant women of second pregnancy, PWFP have inadequate experience and preparation with knowledge of prenatal care, and more likely to have inadequate dietary iron intake.

Area of residence was found not to be associated with anemia in our study. This finding is consistent with results from the *2015 Chinese Chronic Diseases and Nutrition Report* [8]. Although the reasons are yet to be examined for this phenomenon, it provides useful information for future interventions.

Education level was not significantly associated with anemia among pregnant women. Previous studies showed that educational level may influence knowledge and behavior that are important for making health behavior choices [19,20]. However, people with a high education had a more positive attitude toward smoking which might not necessarily translate into health outcomes [21]. In this study, pregnant women with a higher education level did not necessarily have better knowledge about anemia, and better knowledge of anemia did not necessarily translate into health behavioral outcomes. Future anemia interventions should focus on pregnant women of all education levels. 

Physical activity and inactivity have been shown to be associated with obesity and other chronic diseases, and health related quality of life [13,14,15]. However in this study, the average daily exercise time, sleep duration, time spent on TV and computers were not significantly associated with anemia among pregnant women. The reasons are unknown. We hypothesize that young Chinese women are concerned about body image. In general, the prevalence of overweight/obesity among pregnant women in China is low when compared with Western populations. Although overweight/obese was inversely associated with anemia in women in China [13], it may not be the case in young pregnant women.

This study found that compared with those being registered in a low rank hospital, pregnant women who were admitted to medium and high rank hospitals were less likely to have anemia. It could be due to the fact that pregnant women attending high rank hospitals had a high socioeconomic status (income, education and job) and better health literacy. Compared with low rank hospitals, medium and high rank hospitals have more resources and may provide a better prenatal care including health checks and advice. 

We found that women with a medium income had a lower prevalence of anemia than those with a low income (16.7% vs. 10.8%). This is consistent with current knowledge. People with a high income can access more varieties of food and pay more attention to nutrition. Poverty is a known risk factor for anemia in pregnancy. This suggests interventions to reduce anemia among pregnant women should pay more attention to low-income groups.

Compared with non-manual jobs, those with manual jobs or who are unemployed were positively associated with anemia. Manual jobs are often associated with shift work, longer work time, poor work environments and low salary. Shift workers may have different (unfavorable or irregular) dietary habits and patterns. One study also found that staying up late can cause anemia in pregnant women [22]. Special attention should be paid to those with manual jobs or those who are unemployed in the prevention of anemia during pregnancy.

In the study, we found a high prevalence of not eating breakfast (11.0%). However, pregnant women who had breakfast every day were more likely to have anemia than those who skipped breakfast. Cultural beliefs on food taboos for pregnant women contribute to the prevalence of anemia [16]. It could be that some pregnant women ate breakfast but paid less attention to the quality of the breakfast. In China, the main foods consumed for breakfast were grains and vegetables. Studies have found that anemic pregnant women had a higher intake of plant-based foods but less animal-based foods, than those who were not anemic [23]. However, those who skipped breakfast may have snacks, as well as good quality lunches and dinners. This finding may indicate that pregnant women not only need to have breakfast every day, but also need to pay attention to the quality of their breakfast and other meals. In our study, those who had breakfast daily were more likely to have a low income. 

This study has several limitations. Firstly, cross-sectional survey data do not permit reliable inference of causality. Secondly, the face-to-face survey administration design may bring information bias where respondents may not have answered the questions truthfully. However, all questions in the survey were reviewed by a research panel and were tested in a pilot study. Consequently, the questionnaire was less likely to include items that could be perceived as sensitive by study participants. Thirdly, our study was not nationally representative. The sample consisted of pregnant women in five regions of a total of five regions in China, namely Chongqing, Chengdu, Zunyi, Liaocheng, and Tianjin, China. Chongqing, Chengdu, and Zunyi are in south China, Liaocheng and Tianjin are in north China. Furthermore, this study did not distinguish the levels of anemia. Finally, this study did not include information on anemia history. The main concern is the use of self-reported doctor-diagnosed anemia, as the haemoglobin cut-off of anemia set by the Chinese Medical Association is lower than that of WHO. The real prevalence of anemia therefore may be higher than the current finding.

## 5. Conclusions

Pregnant women not eating breakfast are of concern. Pregnant women who eat breakfast every day should pay attention to the quality of their diet. Anemia is highly prevalent among pregnant women. Lower socio-economic statuses, manual jobs, PWFP and those who attend a lower quality hospital for their birth have a greater likelihood of suffering from anemia. Tailored interventions are needed to address these issues. 

## Figures and Tables

**Table 1:** Characteristics of participants stratified by anemia status (*n* = 2345).

Items	Anemia	
	No	Yes
**All participants**	2047 (87.3)	298 (12.7)
**Hospital capacity/quality rank**		
High	1627 (89.20)	197 (10.80)
Medium	262 (84.2)	49 (15.8)
Low	158 (75.2)	52 (24.8)
**Parity**		
PWFP	1520 (86.6)	235 (13.4)
PWSP	527 (89.3)	63 (10.7)
**Single-child status**		
No	1125 (86.6)	174 (13.4)
Yes	922 (88.2)	124 (11.9)
**Status of husband as a single-child**		
No	1010 (86.2)	162 (13.8)
Yes	1037 (88.4)	136 (11.6)
**Age**		
18–25years	531 (85.1)	93 (14.9)
26–35 years	1405 (88.1)	190 (11.9)
36–45 years	111 (88.1)	15 (11.9)
**Marital status**		
Married	1987 (87.3)	288 (12.7)
Unmarried/Divorced/Widowed	60 (85.7)	10 (14.3)
**Education level**		
Low	335 (83.3)	67 (16.7)
Medium	307 (86.7)	47 (13.3)
High	1405 (88.4)	184 (11.6)
**Residence**		
Rural	388 (83.4)	77 (16.6)
Urban	1659 (88.2)	221 (11.8)
**The per capita income of the family**		
Low	509 (83.3)	102 (16.7)
Medium	882 (89.2)	107 (10.8)
High	656 (88.1)	89 (12.0)
**Stages of pregnancy**		
Early pregnancy	254 (86.7)	39 (13.3)
Mid-pregnancy	624 (89.0)	77 (11.0)
Late-stage pregnancy	1169 (86.5)	182 (13.5)
**Job status**		
Inconvenience to the classification of other employee	263 (90.4)	28 (9.6)
Rural migrant workers	96 (81.4)	22 (18.6)
Urban and rural unemployed, half of the unemployed	462 (83.5)	91 (16.5)
Industrial workers of Non-agricultural registered permanent residence	46 (92.0)	4 (8.0)
Individual business	175 (87.9)	24 (12.1)
Business services staff	128 (82.6)	27 (17.4)
Civil servants	366 (92.0)	32 (8.0)
Senior /Middle-level manager in large or medium enterprise	88 (91.7)	8 (8.3)
Private entrepreneur	77 (88.5)	10 (11.5)
Professionals	221 (90.6)	23 (9.4)
Clerk	114 (82.0)	25 (18.0)
Students	11 (73.3)	4 (26.7)
**Breakfast skipping**		
Yes	237 (91.5)	22 (8.5)
No	1810 (86.8)	276 (13.2)
**Average daily exercise time**		
Insufficient	289 (86.5)	45 (13.5)
Sufficient	1758 (87.4)	253 (12.6)
**Sleep duration**		
Insufficient	490 (87.3)	71 (12.7)
Sufficient	1557 (87.3)	227 (12.7)
**Time spent on TV per day**		
<2 h/day	1001 (87.4)	145 (12.7)
≥2 h/day	1046 (87.2)	153 (12.8)
**Time spent on computer per day**		
<2 h	1368 (86.4)	215 (13.6)
2–4 h	413 (88.8)	52 (11.2)
>4 h	266 (89.6)	31 (10.4)
**Smoking status**		
Non-smoker	1965 (87.1)	290 (12.9)
Smoker	82 (91.1)	8 (8.9)

**Table 2:** Odds ratio (95% CI ^(1)^) for anemia according to sociodemographic and lifestyle factors (*n* = 2345).

Parameter		OR ^(2)^	(95% CI)	*p*-Value
**Eat breakfast everyday**				
	No	1	-	-
	Yes	1.89	(1.13, 3.17)	**0.02**
**Parity**				
	PWFP	1	-	-
	PWSP	0.66	(0.48, 0.91)	**0.01**
**Hospital**	**Capacity/quality**			
	Low	1	-	-
	Medium	0.58	(0.37, 0.92)	**0.02**
	High	0.40	(0.28, 0.57)	**<0.00**
**Family income**				
	Low	1	-	-
	Medium	0.68	(0.50, 0.94)	**0.02**
	High	0.90	(0.63, 1.29)	0.58
**Job status**				
	Non-manual	1	-	-
	Manual	1.70	(1.17, 2.45)	**0.01**
	Unemployed	1.42	(1.04, 1.93)	**0.03**
**Stages of pregnancy**				
	Early pregnant women	1	-	-
	Mid-pregnancy women	0.851	(0.54, 1.35)	0.49
	Late-stage pregnant women	1.194	(0.79, 1.81)	0.41

(1) 95% confidence interval (CI); (2) Odds ratio (OR), adjusted for parity, ethnicity, single-child status, status of husband as a single-child, age, marital status, education level, residence, family income, stages of pregnancy, job status, not eating breakfast, average daily exercise time, sleep duration, time spent on TV and computers.

## References

[1] World Health Organization Water-Related Diseases.

[2] Rahman M.M., Abe S.K., Rahman M.S., Kanda M., Narita S., Bilano V., Ota E., Gilmour S., Shibuya K. (2016). Maternal anemia and risk of adverse birth and health outcomes in low- and middle-income countries: Systematic review and meta-analysis. Am. J. Clin. Nutr..

[3] World Health Organization Global Targets 2025. To Improve Maternal, Infant and Young Child Nutrition. http://www.who.int/nutrition/global-target-2025/en/.

[4] World Health Organization (2016). Intermittent Iron and Folic Acid Supplementation in Non-Anaemic Pregnant Women in Malaria-Endemic Areas. http://www.who.int/elena/titles/intermittent_iron_pregnancy_malaria/en/.

[5] Stevens G.A., Finucane M.M., De-Regil L.M., Paciorek C.J., Flaxman S.R., Branca F., Peña-Rosas J.P., Bhutta Z.A., Ezzati M. (2013). Nutrition Impact Model Study Group (Anaemia). Global, regional, andnational trends in haemoglobin concentration and prevalence of total andsevere anaemia in children and pregnant and non-pregnant women for 1995–2011: A systematic analysis of population-representative data. Lancet Glob. Health.

[6] World Health Organization Global Nutrition Targets 2025: Anaemia Policy Brief.

[7] World Health Organization Comprehensive implementation plan on maternal, infant and young child nutrition. Proceedings of the Sixty-Fifth World Health Assembly.

[8] Bureau of Disease Prevention and Control (Office of the National Patriotic Health Campaign Committee) The 2015 Report of Disease Prevention and Control Progress in China. http://en.nhfpc.gov.cn/2015-06/03/c_46242.htm.

[9] Tandu-Umba B., Mbangama A.M. (2015). Association of maternal anemia with other risk factors in occurrence of Great obstetrical syndromes at university clinics, Kinshasa, DR Congo. BMC Pregnancy Childbirth.

[10] Obai G., Odongo P., Wanyama R. (2016). Prevalence of anaemia and associated risk factors among pregnant women attending antenatal care in Gulu and Hoima Regional Hospitals in Uganda: A cross sectional study. BMC Pregnancy Childbirth.

[11] Zhaobin W. (2014). Investigation of anemia among pregnant woman in TianTan Community Health Center area in Beijing in 2013. J. Reprod. Med..

[12] Widyawati W., Jans S., Utomo S., van Dillen J., Janssen A.L. (2015). A qualitative study on barriers in the prevention of anaemia during pregnancy in public health centres: Perceptions of Indonesian nurse-midwives. BMC Pregnancy Childbirth.

[13] Qin Y., Melse-Boonstra A., Pan X., Yuan B., Dai Y., Zhao J., Zimmermann M.B., Kok F.J., Zhou M., Shi Z. (2013). Anemia in relation to body mass index and waist circumference among Chinese women. Nutr. J..

[14] Lacy K.E., Allender S.E., Kremer P.J., de Silva-Sanigorski A.M., Millar L.M., Moodie M.L., Mathews L.B., Malakellis M., Swinburn B.A. (2012). Screen time and physical activity behaviours are associated with health-related quality of life in Australian Adolescents. Qual. Life Res..

[15] Buxton O.M., Marcelli E. (2010). Short and long sleep are positively associated with obesity, diabetes, hypertension, and cardiovascular disease among adults in the United States. Soc. Sci. Med..

[16] Wang L., Xu X., Baker P., Tong C., Zhang L., Qi H., Zhao Y. (2016). Patterns and Associated Factors of Caesarean Delivery Intention among Expectant Mothers in China: Implications from the Implementation of China’s New National Two-Child Policy. Int. J. Environ. Res. Public Health.

[17] Xu X., Rao Y., Abdullah A.S., Sharma M., Guo J.J., Zhao Y. (2016). Preventive behaviours in avoiding indoor secondhand smoke exposure among pregnant women in China. Tob. Control.

[18] Eggleston K., Lu M., Li C., Wang J., Yang Z., Zhang J., Quan H. (2010). Comparing public and private hospitals in China: Evidence from Guangdong. BMC Health Serv. Res..

[19] Mirowsky J., Ross C.E. (1998). Education, personal control, lifestyle and health: A human capital hypothesis. Res. Aging.

[20] Backlund E., Sorlie P.D., Johnson N.J. (1999). A comparison of the relationships of education and income with mortality: The national longitudinal mortality study. Soc. Sci. Med..

[21] Xu X., Liu L., Sharma M., Zhao Y. (2015). Smoking-related knowledge, attitudes, behaviors, smoking cessation idea and education level among young adult male smokers in Chongqing, China. Int. J. Environ. Res. Public Health.

[22] Jin Q.J., Mao L.J. (2015). Anemia in pregnant women and the risk factors. Health Res..

[23] Abdullahi H., Gasim G.I., Saeed A., Imam A.M., Adam I. (2014). Antenatal iron and folic acid supplementation use by pregnant women in Khartoum, Sudan. BMC Res. Notes.

